# Perioperative Pain Management With Bilateral Pecto-intercostal Fascial Block in Pediatric Patients Undergoing Open Cardiac Surgery

**DOI:** 10.3389/fcvm.2022.825945

**Published:** 2022-06-22

**Authors:** Yang Zhang, Jia Min, Shibiao Chen

**Affiliations:** Department of Anesthesiology, First Affiliated Hospital of Nanchang University, Nanchang, China

**Keywords:** Pecto-intercostal Fascial Block, pediatric patients, the length of hospital stay, open cardiac surgery, pain

## Abstract

**Purposes:**

Pediatric open cardiac surgical patients usually suffer from acute pain after operation. The current work aimed to explore the impact of bilateral PIFB in children suffering from open cardiac surgery.

**Methods:**

This work randomized altogether 110 child patients as bilateral PIFB (PIF) and non-nerve block (SAL) groups. This work adopted post-operative pain at exercise and rest statuses as the primary endpoint, whereas time-to-drain removal/extubation/initial defecation, intraoperative/post-operative fentanyl use, and length of ICU and hospital stay as the secondary endpoints.

**Results:**

MOPS were significantly higher at 24-h post-operatively at coughing and rest statuses in SAL group compared with PIF group. Meanwhile, PIF group exhibited markedly lower intraoperative/post-operative fentanyl use amounts, as well as markedly reduced time-to-extubation/initial flatus, and length of ICU/hospital stay.

**Conclusion:**

Bilateral PIFB in pediatric open cardiac surgical patients provide effective analgesia and lower the length of hospital stay.

## Introduction

Pediatric patients receiving open cardiac surgery always experience acute post-operative pain, with median sternotomic incision being the main pain source (1). Insufficient post-operative analgesia may lead to prolonged immobilization, hypertension, hypoxia, pulmonary hypertensive crisis, tachyarrhythmia, the inability to cough due to median sternotomy, increased length of intensive care unit (ICU) stay and length of hospital stay (2). Non-invasive and invasive methods are used in children suffering from open cardiac surgery for post-operative pain management, such as intravenous opioids, thoracic epidural block (TEA), paravertebral block techniques and the transversus thoracis muscle plane (TTMP) block (3, 4). Intravenous opioids for acute pain induce adverse reactions, such as sufficient sedation, respiratory depression, ileus, post-poned tracheal extubation, immunosuppression, nausea/vomiting, drowsiness, and cough suppression (5).

Paravertebral block techniques and TEA provided effective analgesia in pediatric cardiac surgical patients (3). But hypotension resulting from sympathectomy, pneumothorax, block failure, and adverse reactions like epidural hematoma following infection or complete heparinization, and spinal cord injury greatly restrict their application in cases receiving cardiac surgeries (6). TTMP block could be used in pediatric cardiac surgery for post-operative pain (7), but there was a risk of vascular damage and pneumothorax.

The ultrasound-guided Pecto-intercostal Fascial Block (PIFB) was a newly developed technique and has been used in cardiac surgery (8). To the best of our knowledge, there was no randomized controlled trial (RCT) of PIFB block for pediatric cases who underwent open cardiac surgical resection. Therefore, this work focused on assessing the effect of bilateral PIFB on analgesia as well as reducing hospital stay of patients receiving cardiac surgical treatment.

## Methods

The Ethics Committee of First Affiliated Hospital of Nanchang University approved our study protocols. This work was registered at the Chinese Clinical Trial Registry (ChiCTR; registration number ChiCTR 2000030609). The parents of child patients were informed of the study protocols, and they provided informed consents for participation.

The present double-blind RCT was carried out on children between the age groups of 2 and 6 years who experienced elective open heart operation *via* median sternotomy. The exclusion criteria in our trial was as follows: congestive heart failure, allergic to ropivacaine, hepatic or renal failure, psychiatric problems, redo surgery and pre-operative inotropic support. All cases were randomized as PIF and SAL groups to receive bilateral PIFB using 0.2% ropivacaine and saline at the identical volume, respectively.

### Surgery and Anesthesia

Pre-medication was given 30 min before anesthesia with oral midazolam (1 mg/kg, 20 mg at most) in each case. Patients' electrocardiography (ECG), pulse oximetry, invasive arterial blood pressure (BP), central venous pressure, central temperature, and end-tidal carbon dioxide were monitored when they entered the operating room. General anesthesia was triggered with midazolam 0.05 mg/kg, fentanyl 6–10 μg/kg, rocuronium 0.6 mg/kg and etomidate 0.3 mg/kg, followed by the implementation of endotracheal intubation. In addition, fentanyl, rocuronium, and propofol were applied for anesthesia maintenance, with BIS being kept at 45–55 in each case. Intravenous fentanyl and acetaminophen were used to perform post-operative analgesia in our study. The same surgeons at our institution were responsible for performing all surgical procedures. Post-operatively, each child patient was set to cardiac surgery ICU according to pre-determined schedule.

### Randomization and Blinding

The random number table method was utilized to randomize all cases as PIF and SAL groups for 0.2% ropivacaine and saline treatment, separately. The allocation of different groups was maintained within a sealed envelope. Ropivacaine and saline, which looked the same, were prepared by a nurse at the post-anesthesia care unit. Thereafter, the as-prepared solution was injected in the pecto-intercostal fascial plane by an experienced anesthesiologist who was blind to group allocation in a 20-min period. Post-operative data were collected *via* a third reviewer blind to grouping. Surgeons, cases, anesthesiologist, nurses, ICU staff, or additional reviewers were blind to medication assignment in the present double-blind RCT.

### Ultrasound-Guided PIFB

By adopting the high-frequency linear ultrasound probe (Mindray, Shenzhen, China), this work carried out PIFB in the supine position. In brief, a probe was inserted at 2 cm lateral from and parallel to the sternum. Thereafter, the external intercostal muscle, pectoralis major muscle, lungs, pleura, and costal cartilage were found. Later, after the localization of pecto-intercostal fascial plane between costal cartilage or external intercostal muscle and pectoralis major muscle, by adopting the in-plane approach, this work inserted a 22-G needle (diameter, 70 mm; Tuoren, Henan, China) beneath pectoralis major while on the top of external intercostal muscle. In the meantime, the correct placement of the needle tip was determined by injecting 1 ml saline. At last, this work injected 0.2% ropivacaine (1.5 mg/kg) into the plane at two positions, over the second and fourth ribs. The identical approach was applied for contralateral PIFB process.

### Study Parameters

Post-operative pain was set as the primary endpoint in this work, which was measured at 3/6/9/12/24/48-h post-extubation at exercise (coughing) and rest statuses, as determined through Modified Objective Pain Score (MOPS) (9). Meanwhile, time-to-drain removal/extubation/first defecation, intraoperative/post-operative fentanyl use, length of ICU/hospital stay, together with potential PIFB complications were deemed as the secondary endpoints.

### Statistical Analysis

Sample size in this work was determined according to one preliminary research (*n* = 12/each) comparing pain scores post-operatively. Therefore, for achieving the type-I, type-II error and power of α = 0.05, β = 0.1 and 90%, separately, we needed 92 cases, with a dropout rate of 20%. At last, this work required 110 child patients altogether.

SAS software (version 9.1.3, North Carolina, USA) was adopted for statistical analysis. Continuous data between both groups were compared by Mann-Whitney *U-*test and student *t-*test. At the same time, Fisher's exact or Chi-square test was applied in comparing qualitative data. This work also compared MOPS post-extubation between two groups by repeated-measures (two-way) ANOVA. *P* < 0.05 indicated statistical significance.

## Results

**Figure 1:**
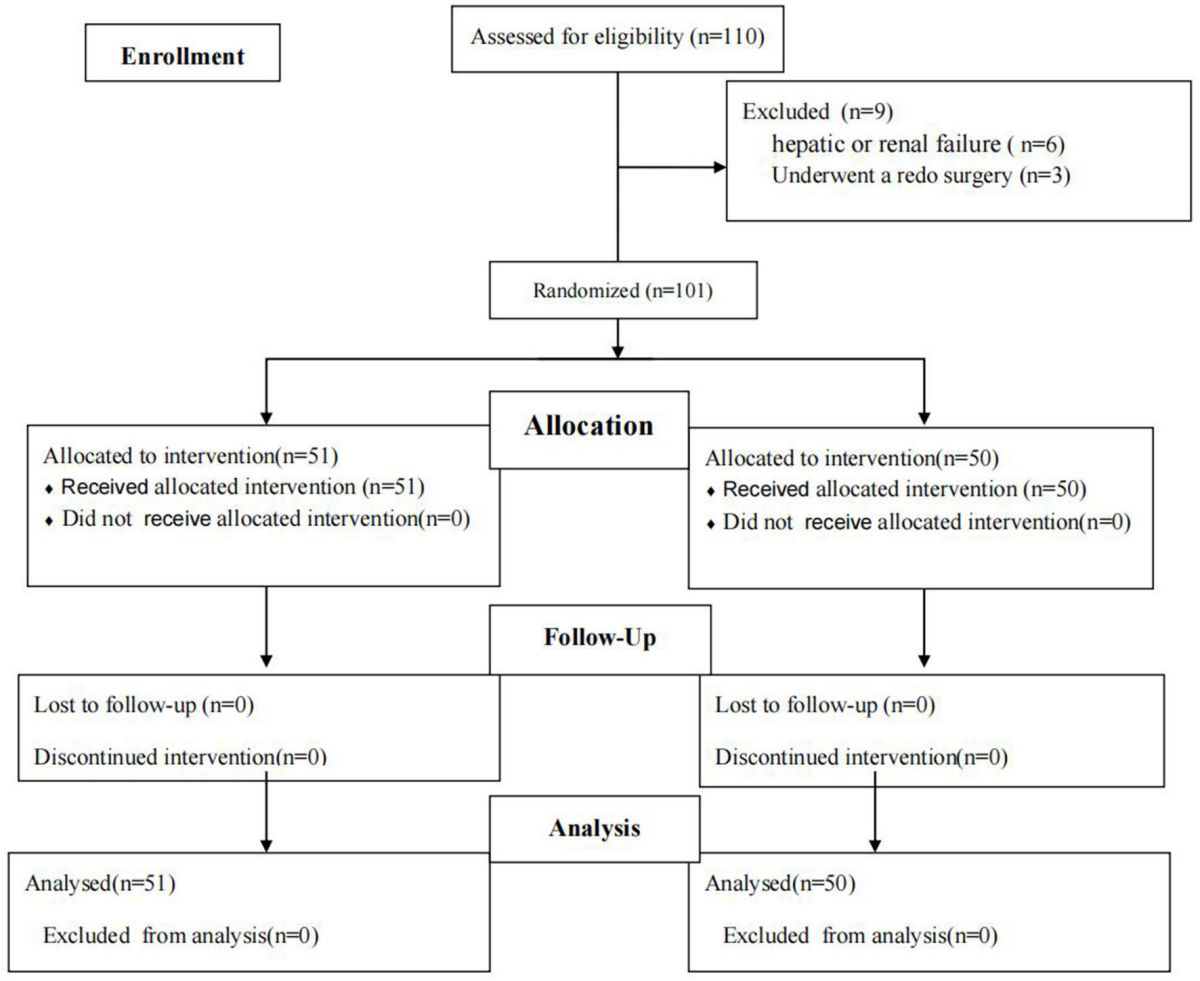
Patient flow diagram.

One hundred and 10 children were randomized (55 in per group). This work eliminated four and five cases from PIF and SAL groups, separately due to redo surgery (*n* = 3) as well as hepatic or renal failure (*n* = 6). Finally, 101 children were left in our study for subsequent analyses, with 51 in the PIFB and 50 in the SALI groups (Figure 1). Baseline features of patients during surgery were comparable between two groups (Table 1). No complications of PIFB were occurred in our patients.

**Table 1:** Demographic data and surgical procedures.

	**PIF group (*n* = 51)**	**SAL group (*n* = 50)**	***P*-value**
Age (years)	4.2 ± 1.6	3.9 ± 1.9	0.65
Weight (Kg)	16.8 ± 6.4	16.2 ± 7.3	0.59
ASA classification (II/III)	39/12	35/15	0.62
Sex (male/female)	30/21	26/24	0.71
Duration of surgery (min)	83.6 ± 15.5	88.7 ± 19.3	0.58
Cardiopulmonary bypass time (min)	32.9 ± 10.7	35.8 ± 12.1	0.67
Surgical procedures			0.72
ASD closure	27	24	
VSD closure	24	26	

**Figure 2:**
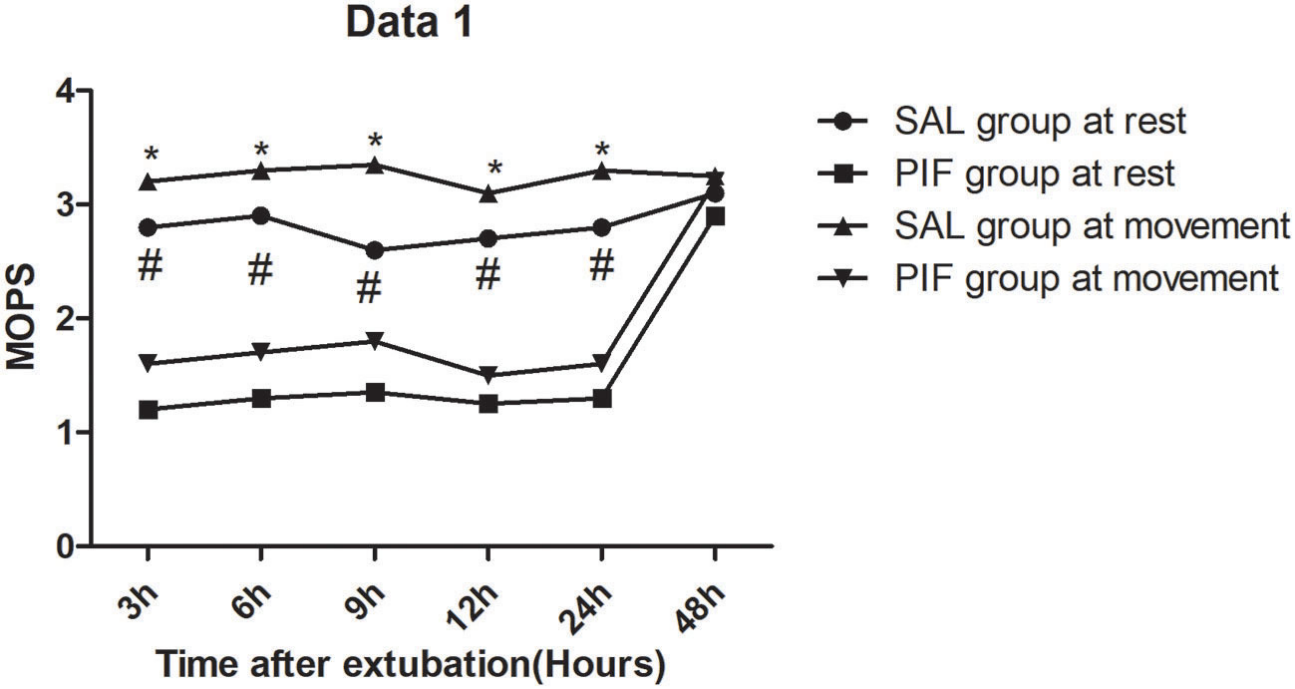
Pain intensity at rest after extubation which was measured by Modified Objective Pain Score (MOPS). #*P* < 0.05 considered statistically significant. Pain intensity at movement after extubation which was measured by Modified Objective Pain Score (MOPS). *P < 0.05 considered statistically significant.

Modified objective pain score markedly increased at 3/6/9/12/24-h post-extubation at exercise (coughing) and rest statuses, and difference at 48-h post-extubation were not significant between two groups (Figure 2). The PIF group reported significantly less intraoperative/post-operative fentanyl use (Table 2), along with markedly reduced time-to-extubation/first flatus, and length of ICU/hospital stay (Table 2). Difference in time-to-drain removal was not significant between two groups (Table 2).

**Table 2:** Intra- and post-operative clinical outcomes.

	**PIF group (*n* = 51)**	**SAL group (*n* = 50)**	***P*-value**
Intraoperative fentanyl consumption (μg/kg)	13.3 ± 1.7	19.9 ± 2.2	<0.01
Post-operative fentanyl consumption (μg/kg)	1.4 ± 0.4	3.8 ± 1.3	<0.01
Time to extubation (min)	33.2 ± 9.7	75.7 ± 9.6	<0.01
Time to drain removal (h)	45.5 ± 8.5	47.3 ± 7.9	0.57
Length of stay in the ICU (h)	9.8 ± 2.3	19.9 ± 5.3	<0.01
Time to first feces (h)	30.6 ± 5.7	39.7 ± 6.4	<0.05
Length of hospital stay (d)	6.9 ± 0.9	8.1 ± 0.9	<0.01

## Discussion

This RCT illustrated the applying bilateral PIFB under ultrasonic guidance in pediatric patients undergoing open cardiac surgery offered efficient analgesia till 24-h post-extubation, while reducing fentanyl use perioperatively. Meanwhile, this technique decreased time-to-extubation/first flatus, along with length of hospital/ICU stay.

High-dose fentanyl is usually adopted in the infusion regimen or as bolus dosing for the anesthesia of cardiac surgery in child patients, which can attain stable hemodynamics and efficient post-operative analgesia (10). Nonetheless, high-dose opioids may induce side reactions such as pruritus, respiratory depression, ileus, post-poned recovery, extended ventilation (11), along with longer ICU/hospital stays (12). According to our results, bilateral PIFB under ultrasonic guidance substantially decreased perioperative fentanyl use with no side reactions. After surgery, PIF group had markedly reduced mean time-to-extubation relative to SAL group, while the reduced fentanyl use perioperatively might be its major cause. The shorter ICU stay length might be possibly associated with the efficient analgesic effect induced by bilateral PIFB under ultrasonic guidance, significantly reduced fentanyl used and shorter time-to-extubation post-operatively. Consequently, decreasing fentanyl use might lay the foundation to promote recovery after open cardiac surgery.

The PIFB offered efficient analgesia for thymectomy *via* median sternotomy (13, 14) and cardiac surgery (8). Moreover, several articles have described PIFB in sternal fracture pain (15), rib cage pain in ICU patients (16), breast surgery (17), and the subcutaneous-implantable cardioverter defibrillator system implantation (18). As far as we know, the present double-blind RCT first identified the effect of bilateral PIFB on providing efficient pain relief perioperatively for child patients receiving cardiac surgical treatment without adverse events. Bilateral transversus thoracic muscle plane (TTMP) block under ultrasonic guidance can also be adopted for cardiac surgical treatment in child patients for post-sternotomy pain (7). The advantages of PIFB over TTMP block in those suffering from open cardiac surgery were as follows (4, 19). First, both internal mammary vein and artery penetrated TTMP, which was associated with the risk of vascular laceration when blocking. Second, transversus thoracic muscle is usually very thin, especially in children. Therefore, the transversus thoracic muscle, which is also very close to the pleura, can hardly be visualized by ultrasonography (20). That's why there's a risk of pneumothorax in TTMP block. Third, in patients with previous chest surgery, the scar tissue would influence the spread of local anesthetic (21).

Pediatric open cardiac surgical patients usually suffered from extended and serious post-operative pain, in particular in median sternotomic incision (22). Poorly controlled post-operative pain in pediatric open cardiac surgical patients could lead to destructive outcomes like tachyarrhythmia, pulmonary hypertensive crisis, hypertension, extended immobilization, longer ICU/hospital stay length, and hypoxia (2). Our study demonstrated that the ultrasound-guided PIFB provided effective analgesia for 24 h after extubation to children. PIFB shows a decreased risk and invasive degree compared with TTMP block, paravertebral nerve block and thoracic epidural analgesia due to concern of epidural or spinal hemorrhage and hematoma, pneumothorax, vascular laceration and hypotension. And PIFB could reduce the sympathetic excitement and opioid consumption perioperatively, so it could reduce time to first flatus in our study. Therefore, PIFB represented the new, efficient and safe technology for local analgesia in child cases who received open cardiac surgical treatment.

Finally, bilateral PIFB in pediatric open cardiac surgical patients could reduce perioperative consumption of fentanyl, decreased the time-to-extubation/first flatus as well as ICU stay length. The above findings lay the foundation for decreasing hospital stay length.

This study was limited by the uncertain optimal volume and concentration and the short duration impairing the ability to assess long-term post-operative chronic pain.

And, Continuous PIFB block may provide persistent post-operative analgesia for the median sternotomy, but the technology was not utilized in the present work. In further study, we should gradually solve these three problems for pediatric cardiac surgery patients.

In conclusion, our study demonstrated that ultrasound-guided bilateral PIFB in pediatric open cardiac surgical patients shortens hospital stay length through offering the efficient post-operative analgesia, which has substantially reduced the use of fentanyl perioperatively, decreased the time-to-extubation/first flatus as well as ICU stay length.

## Data Availability Statement

The raw data supporting the conclusions of this article will be made available by the authors, without undue reservation.

## Ethics Statement

The studies involving human participants were reviewed and approved by the Ethics Committee of First Affiliated Hospital of Nanchang University and it was registered in the Chinese Clinical Trial Registry (ChiCTR) (registration number ChiCTR 2000030609). Written informed consent to participate in this study was provided by the participants' legal guardian/next of kin.

## Author Contributions

YZ, JM, and SC: study design/planning and data analysis. YZ and JM: study conduct. YZ and SC: writing the manuscript. All authors revised the manuscript.

## Funding

The project was funded by Department of Science and Technology of Jiangxi Province [20203BBGL73195] and [20212BAG70034].

## Conflict of Interest

The authors declare that the research was conducted in the absence of any commercial or financial relationships that could be construed as a potential conflict of interest.

## Publisher's Note

All claims expressed in this article are solely those of the authors and do not necessarily represent those of their affiliated organizations, or those of the publisher, the editors and the reviewers. Any product that may be evaluated in this article, or claim that may be made by its manufacturer, is not guaranteed or endorsed by the publisher.
